# Challenges of applying multicellular tumor spheroids in preclinical phase

**DOI:** 10.1186/s12935-021-01853-8

**Published:** 2021-03-04

**Authors:** Se Jik Han, Sangwoo Kwon, Kyung Sook Kim

**Affiliations:** 1 Department of Biomedical Engineering, Graduate School, Kyung Hee University, Seoul, 02447 Korea; 2 Department of Biomedical Engineering, College of Medicine, Kyung Hee University, Seoul, 02447 Korea

**Keywords:** Multicellular tumor spheroids, Drug screening, Therapeutic efficacy, High-throughput, Microfluidics

## Abstract

The three-dimensional (3D) multicellular tumor spheroids (MCTs) model is becoming an essential tool in cancer research as it expresses an intermediate complexity between 2D monolayer models and in vivo solid tumors. MCTs closely resemble in vivo solid tumors in many aspects, such as the heterogeneous architecture, internal gradients of signaling factors, nutrients, and oxygenation. MCTs have growth kinetics similar to those of in vivo tumors, and the cells in spheroid mimic the physical interaction of the tumors, such as cell-to-cell and cell-to-extracellular matrix interactions. These similarities provide great potential for studying the biological properties of tumors and a promising platform for drug screening and therapeutic efficacy evaluation. However, MCTs are not well adopted as preclinical tools for studying tumor behavior and therapeutic efficacy up to now. In this review, we addressed the challenges with MCTs application and discussed various efforts to overcome the challenges.

## Introduction

Recently, the three-dimensional (3D) multicellular tumor spheroids (MCTs) model has been gaining increased recognition as an intermediate step between in vitro and in vitro models, thus offering enhanced biological relevance in research fields, such as tumor biology and drug screening [1–3]. MCTs are cell clusters formed by either self-assembly or forced growth starting from single-cell suspensions. The cells are closely packed with high density in spheroids. Therefore, the cells in MCTs communicate strongly and sustain complex communication between cells and extracellular matrix (ECM) [4].

MCTs formation can be achieved with 3D scaffold incorporation or in scaffold-free conditions. In the scaffold-based approach, the cells are seeded on an acellular 3D artificial matrix that mimics ECM architecture [5]. The most frequently used methods in scaffold-free conditions are liquid overlay and hanging drop methods. Cells can aggregate due to the low adhesive surface in the liquid overlay method, and surface tension and gravity are instrumental in forming a spheroid in the hanging drop method [6, 7]. Recent advances in bioengineering techniques have contributed to the development of the spheroid culture system by employing microfluidic channels and 3D bio-printing [8–10].

MCTs closely mimic in vivo solid tumors' main features, such as structural organization and the gradients of oxygen, pH, and nutrients [11, 12]. Beyond a critical size of about 500 µm, MCTs show characteristic features of avascular tumors with an external proliferating zone, an internal quiescent zone, and a necrotic core due to gradients of nutrient and oxygen concentration [13–18]. Besides, MCTs revealed similarity to in vivo solid tumors in growth kinetics, metabolic rates, and resistance to chemotherapy and radiotherapy [19–21]. MCTs' physiological relevance with in vivo solid tumors has contributed to advancing our understanding of tumor biology, such as proliferation, invasion, metastasis, and vascularization [22–25]. Also, it provides new preclinical models for the development of anticancer drug and therapeutic approaches, including radiotherapy and immunotherapy.

Organoids are another type of multi-cellular 3D structures. MCTs are cell aggregates typically composed of cancer cells cultured under scaffold-based or -free conditions. Unlike MCTs, organoids are comprised of organ-specific cells derived from primary tissue or stem cells capable of self-renewal, self-organization and exhibit organ functionality [26, 27]. A scaffolding extracellular environment such as Matrigel and collagen is used to support the developing microstructure architecture of organoids. Organoids are also employed in cancer research because they can provide insight into the cancer biology by imitating human tumors' pathophysiology.

**Fig. 1:**
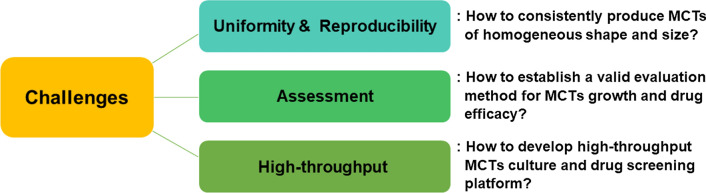
Several issues of applying MCTs at a preclinical level for screening of new anticancer drugs and development of treatment

In this review, we primarily focused on the issues raised in MCTs application. Despite its excellent properties, several issues remain in using MCTs in the preclinical phase, such as developing and screening new anticancer drugs (Fig. 1). The first issue is uniformity and reproducibility in consistently producing MCTs of homogeneous shape and size. The second issue is assessing how to establish a valid evaluation method for MCTs growth and drug efficacy. The third issue is regarding high-throughput methods. The development of high-throughput MCTs culture and drug screening methods is an essential requirement for commercial applications. We addressed the three issues and summarized the efforts to address these issues.

## Uniformity and reproducibility

The physiological characteristics of a spheroid culture of cells growing in a 3D environment can differ considerably from those of cells in a 2D monolayer. The cells in MCTs formed strong interaction between cells and between cells and their environments; this significantly affected spheroid formation. In addition, MCTs formation is linked to various factors, such as cell type, culture technique, medium composition and volume, and cell density. These factors cause variability in MCTs formation, resulting in difficulties in reproducible spheroid formation.

### MCTs growing and structure

**Fig. 2:**
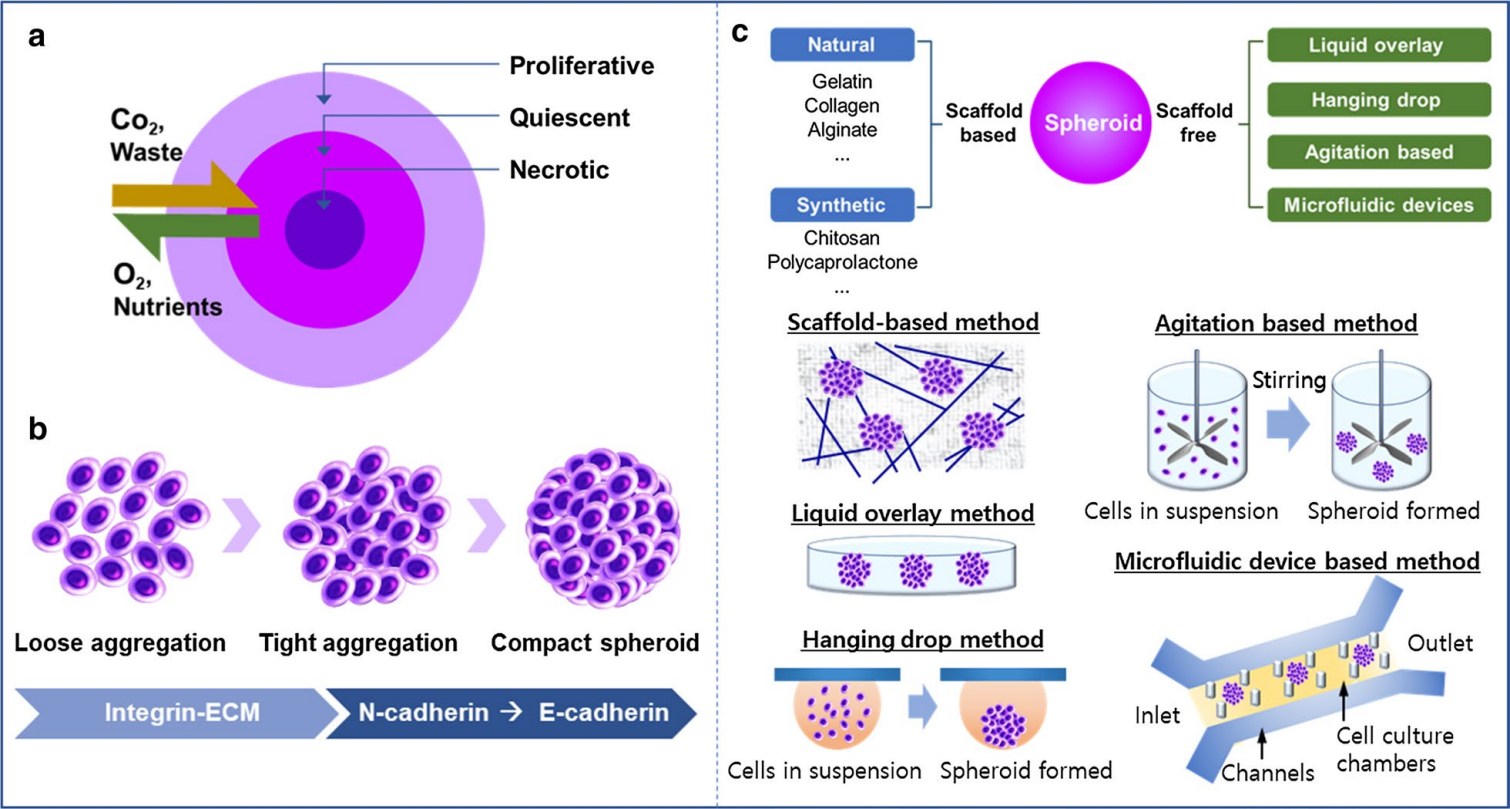
**a** Structure of MCTs, which is organized in three main layers, including a proliferative outer layer, quiescent inner layer, and necrotic core. MCTs have a gradient in oxygen, carbon dioxide, and nutrient content similar to in vivo solid tumors. **b** MCTs formation process. Cells initially aggregate by loose bonds between integrin and ECM and then form close contact through N-cadherin-to-E-cadherin interactions. **c** MCTs culture methods which are categorized in two groups—scaffold-based and scaffold-free cell culture methods. Several techniques are developed in each group

The MCTs can be cultured with only cancer cells or co-cultured with cancer cells and other cell types, such as fibroblasts, endothelial cells, or immune cells [28, 29]. Once the cells are seeded, cells aggregate and form a spherical shape within several days [30]. Like in vivo solid tumors, MCTs have heterogeneous cell populations and pathophysiological gradients (Fig. 2a). There are proliferating cells on the outer layer, quiescent cells on the inner layer, and necrotic cells in the spheroid's core [31, 32]. These heterogeneous cell layers result from limited diffusion of oxygen and nutrients into the sphere. The cells on the outer layer are highly proliferative owing to more accessible access to oxygen and nutrients. Moving toward the center, the supply of oxygen and nutrients decreases, and the amount of carbon dioxide and waste increases [33, 34]. Therefore, cells present in the core of the spheroid remain in a senescent or necrotic state.

### Morphology of MCTs

MCTs start to form after few days of seeding cells on the substrate with suppressed adhesion. They are irregularly round-to-oval bodies in the early stages of formation and then assume a complete spheroid shape as the culture progresses. The morphology of MCTs is influenced by various factors, such as the cell type, cell density, culture media, culture method, and mechanical stress [35]. MCTs morphology can be classified into three groups by their compactness: compact spheroids, tight aggregates, and loose aggregates of cells (Fig. 2b) [36, 37]. Cells are tightly bound to each other in compact spheroids, making it challenging to distinguish single cells. Cells in tight or loose aggregation cannot form a complete sphere and are easily disintegrated. The initial aggregation of cells is initiated by integrin-mediated attachment to ECM molecules, and the cells are aggregated compactly by E-cadherin mediation [34].

#### MCTs morphology depending on cell type

To date, the suitability of MCTs formation has been investigated in several hundred cancer cells. Some cancer cells showed high efficiency of spheroid formation, whereas others showed low efficiency or none at all. Even for the same tumor type, the efficiency of MCTs formation was different depending on cell lines. The MCF-7, BT-474, T47D, and MDA-MB-361 breast cancer cell lines formed compact spheroids (CS), whereas other cell lines aggregated tightly (TA, MDA-MB-435S) or loosely (LA, MDA-MB-231, MDA-MB-468, and SK-B-3) [36]. The gastric cancer cells cultured in the same condition also formed a spheroid or aggregated depending on cell lines [38]. Cell lines of RF-1, RF-48, and Hs-746 T formed compact spheroids; MKN-28, MKN-74, and N87 formed tight aggregates; and SNU-5 and SNU-6 formed loose aggregates. More classification of MCTs morphology depending on cell types is listed in Table 1. The inherent differences in cell-to-cell adhesions of different cancer cell lines result in differences in the formation and compactness of their spheroids. The cell lines that formed compact spheroids expressed a high E-cadherin level, whereas tight aggregates showed accelerated expression of N-cadherin [36]. When cells lose the adhesion molecules, they also lose the ability to aggregate into a sphere.

**Table. 1.** MCTs formation depends on the cell type

Tumor type	Cell line	MCTs morphology	Culture conditions (media, peroid, technology)	References
Breast cancer	MCF-7	CS	RPMI + 10% FCS + L-Glutamine, 24 h, low adhesion plate	[22]
	BT-474	CS	Sodium pyruvate + HEPES, 24 h, low adhesion plate	
	T-47D	CS	RPMI + 10% FCS + L-Glutamine, 24 h, low adhesion plate	
	MDA-MB-361	CS	RPMI + 10% FCS + L-Glutamine, 24 h, low adhesion plate	
	MDA-MB-435S	TA	RPMI + 10% FCS + L-Glutamine, 24 h, low adhesion plate	
	MDA-MB-231	LA	RPMI + 10% FCS + L-Glutamine, 24 h, low adhesion plate	
	MDA-MB-468	LA	RPMI + 10% FCS + L-Glutamine, 24 h, low adhesion plate	
	SK-BR-3	LA	McCoy's 5A, 24 h, low adhesion plate	
	MCF-7	CS	RPMI + 25% methocel, 3 days, hanging drop	[4]
	MDA-MB-231	TA	RPMI + 25% methocel, 3 days, hanging drop	
	SK-BR-3	LA	RPMI + 25% methocel, 3 days, hanging drop	
Colon cancer	HCT116	CS	High glucose, 4 days culture low adhesion plate	[39]
	DLD-1	TA	DMEM, 4 days culture low adhesion plate	
	SW620	LA	RPMI, 4 days, low adhesion plate	
Gastric cancer	RF-1	CS	RPMI, 2 days, liquid overlay technique	[38]
	RF-48	CS	RPMI, 2 days, liquid overlay technique	
	Hs-746 T	CS	RPMI, 2 days, liquid overlay technique	
	MKN-28	TA	RPMI, 2 days, liquid overlay technique	
	MKN-74	TA	RPMI, 2 days, liquid overlay technique	
	N87	TA	RPMI, 2 days, liquid overlay technique	
	SNU-5	LA	RPMI, 2 days, liquid overlay technique	
	SNU-16	LA	RPMI, 2 days, liquid overlay technique	
Head and neck cancer	FaDu	CS	RPMI + 10% FCS, 72 h, low adhesion plate	[40]
	HLaC78	CS	RPMI, 2 days, liquid overlay technique	
	HSmC78	CS	RPMI, 2 days, liquid overlay technique	
	Cal27	CS	RPMI, 2 days, liquid overlay technique	
	PE/CA-PJ41	CS	RPMI, 2 days, liquid overlay technique	
	HNO210	CS	DMEM, 72 h, low adhesion plate	
	SCC4	CS	DMEM/F12, 72 h, low adhesion plate	
	FaDu	TA	RPMI, 2 days, liquid overlay technique	
	Hep2	TA	MEM, 72 h, low adhesion plate	
	HPaC79	LA	RPMI, 2 days, liquid overlay technique	
	HLaC79	LA	RPMI, 2 days, liquid overlay technique	
	HLaC79-tAX	LA	RPMI, 2 days, liquid overlay technique	
Glioblastoma	U-87 MG	CS	DMEM, 24–48 h, low adhesion plate	[41]
	A172	CS	DMEM, 24–48 h, low adhesion plate	
	SF126	CS	DMEM, 24–48 h, low adhesion plate	
	LN-229	CS	DMEM, 24–48 h, low adhesion plate	
Neuroblastoma	SH-SY5Y	CS	RPMI, 24–48 h, low adhesion plate	[41]
	KELLY	CS	RPMI, 24–48 h, low adhesion plate	
	SHEP	TA	RPMI, 24–48 h, low adhesion plate	
	IMR-32	TA	DMEM, 24–48 h, low adhesion plate	
Pancreatic	PANC-1	CS	DMEM, 24–48 h, low adhesion plate	[41]
	MIA PaCa-2	LA	DMEM, 24–48 h, low adhesion plate	

*CS* compact spheroids, *TA* tightly aggregated, *LA* loosely aggregated

#### MCTs formation depending on culture methods

There are several methods to generate MCTs, which are categorized in two groups: scaffold-based and scaffold-free cell cultures (Fig. 2c). In scaffold-based culture, the cells are seeded on a 3D artificial matrix or dispersed on the hydrogel. Since the scaffold mimics the ECM, it provides mechanical support and offers cell-to-ECM interaction opportunities [42, 43]. The scaffold can be produced with various biomaterials, including natural and synthetic compositions. Natural polymers, such as gelatin, alginate, collagen, and Matrigel, are preferred because of their biocompatibility and formability [44–48]. Or, the synthetic polymers, such as poly (lactic-co-glycolic) acid (PLGA) or polycaprolactone (PCL), and poly (ethylene glycol) (PEG), are used in 3D scaffold fabrication. The synthetic polymers provide abundant availability; they can be produced in large uniform quantities and tailored for specific applications [49–52].

In a scaffold-free culture, four major techniques are available for spheroid formation, including agitation-based technique, liquid overlay technique, hanging drop technique, and microfluidic technique. In the agitation‐based technique, cells aggregate under continuous stirring to prevent the cell from adhering to surfaces [37, 43]. The hanging drop technique uses the surface tension of a cell liquid drop suspended on a glass coverslip and gravity to induce aggregation and accelerate spheroid formation [53]. In the liquid overlay technique, cells are seeded on non-adhesive surfaces to avoid cell attachment [21]. Super-hydrophobic agar or agarose are frequently applied to make non-adherent surfaces [43]. Microfluidics has been widely investigated as a promising technique because it can offer 3D environments, long-term culture, and precise handling of spheroids [54, 55]. Among the above-mentioned techniques, agitation‐based technique, hanging drop technique, and liquid overlay technique are easy and cheap to operate with no specialized equipment needed. Conversely, microfluidics offers the scale‐up of spheroid formation under precisely controlled conditions, making it suitable for high‐throughput screening [20, 43]. Both agitation‐based and liquid overlay techniques need optimization to form MCTs with uniform size and morphology, and the hanging drop technique is labor- and time-consuming.

Choosing a MCTs formation technique is very important because they are not equivalently working to form spheroids (Table 2). During the same culture period, MCF-7 and MDA-MB-231 spheroids created using agitation‐based (nutator) and hanging drop techniques grew larger than those created using the liquid overlay technique [56]. In addition, the MCTs generated using agitation-based and hanging drop techniques revealed higher collagen type I levels than those created using the liquid overlay technique. In the liquid overlay technique, the degree of MCTs formation depends on medium additives (25% methocel, 25% methocel + 1% Matrigel or 3.5% Matrigel) [4]. Bladder cancer cells (RT4) can form compact spheroids with both hanging drop and liquid overlay techniques; however, the growth rate of spheroids relative to cell seeding density is better in the liquid overlay technique [57]. Taken together, it seems that the hanging drop technique is more effective than the liquid overlay technique for forming highly compact tumor spheroids in certain cell types. Therefore, it is necessary to establish standardized and reproducible protocols for MCTs formation with comparable size and morphology.

**Table. 2.** MCTs formation depends on culture methods

Tumor type	Cell line	Culture technology				
		Agitation‐based	Liquid overlay	Hanging drop	Suspension	References
Breast cancer	MCF-7	Small spheroid High collagen content	Large spheroid Small collagen content	Small spheroid High collagen content	–	[56]
Ovarian cancer	OVCAR8	Small spheroid High collagen content	Large spheroid Small collagen content	Small spheroid High collagen content	–	[56]
Breast cancer	MCF-7	–	Large spheroid	Large or small spheroid	Large or small spheroid	[4]
	MDA-MB-231	–	Loose aggregation	Tight aggregation	No aggregation	
	SK-BR-3	–	Loose/no aggregation	Loose aggregation	No aggregation	
Bladder cancer	RT4	–	Large spheroid	Small spheroid	–	[57]
Head and neck cancer	Cal27	–	Large spheroid	Small spheroid	–	[58]
	FaDu	–	Large spheroid	Small spheroid	–	
	PiCa	–	Large spheroid	Small spheroid	–	

#### Morphologic and ultrastructural characterization

The overall development of MCTs is monitored during the experiment conventionally using optical microscopy. Images of MCTs are captured at the desired time points to analyze the spheroid volume growth kinetics. Optical microscopy images also provide morphologic information about MCTs. However, given the light wavelength-related limitations of an optical microscope’s resolution, an electron microscope is used for precise imaging-based analyses of MCTs. The scanning electron microscope (SEM), a type of electron microscope, is a widely used method to characterize material surface in micro-and nanometer-scaled resolutions. For SEM imaging, the MCTs should be fixed, dehydrated, and then coated with conducting materials, such as gold–palladium [59]. The SEM images provide precise morphologic details of MCTs, with the visualization of cell-clustering and clear periphery [59, 60].

The transmission electron microscope (TEM) is used to analyze the internal structure of MCTs. TEM imaging entails the transmission of a beam of electrons through an ultrathin sample; hence, it provides information on the internal structure of the sample and yields high resolution [61]. The sample for TEM imaging should also be fixed and dehydrated and then sectioned into a thin slice (approximately 70 nm) before it is coated with a conducting material [59]. TEM is very useful in analyzing the internal structure of MCTs and the drug delivery process. The TEM images of the SUM1315 and MDA-MB-231 spheroids showed adjoined cells with intact plasma and nuclear membranes and two types of cell junctions, including tight junctions and anchoring junctions [59]. TEM has been used to visualize the penetration behavior of anticancer therapeutics, such as doxorubicin, quantum dots, and micelles, and to monitor their internalization into cells [34, 62].

### Size determinant of MCTs

MCTs size is a critical parameter related to tumor biology and drug screening; it is mainly determined by the cell type, culture time, and seeding density. The heterogeneous cell layers depend on the MCTs size, and the delivery of nutrients and oxygen inside the spheroid becomes more difficult as the spheroid becomes larger. Therefore, optimizing or controlling the MCTs size is desired in an application, but it remains challenging. Although the size depends on some parameters, the MCTs that form are often very different in size, even under the same conditions. For example, when lung cancer cells were grown on an alginate scaffold over 13 days, they formed spheroids in a size range of 100–300 µm [63]. The human colorectal cancer cell line HT-29 spheroids grown on confined pillar structure for four days also showed the distribution in a size range of 70–180 μm with an average size of 110 μm [64].

**Fig. 3:**
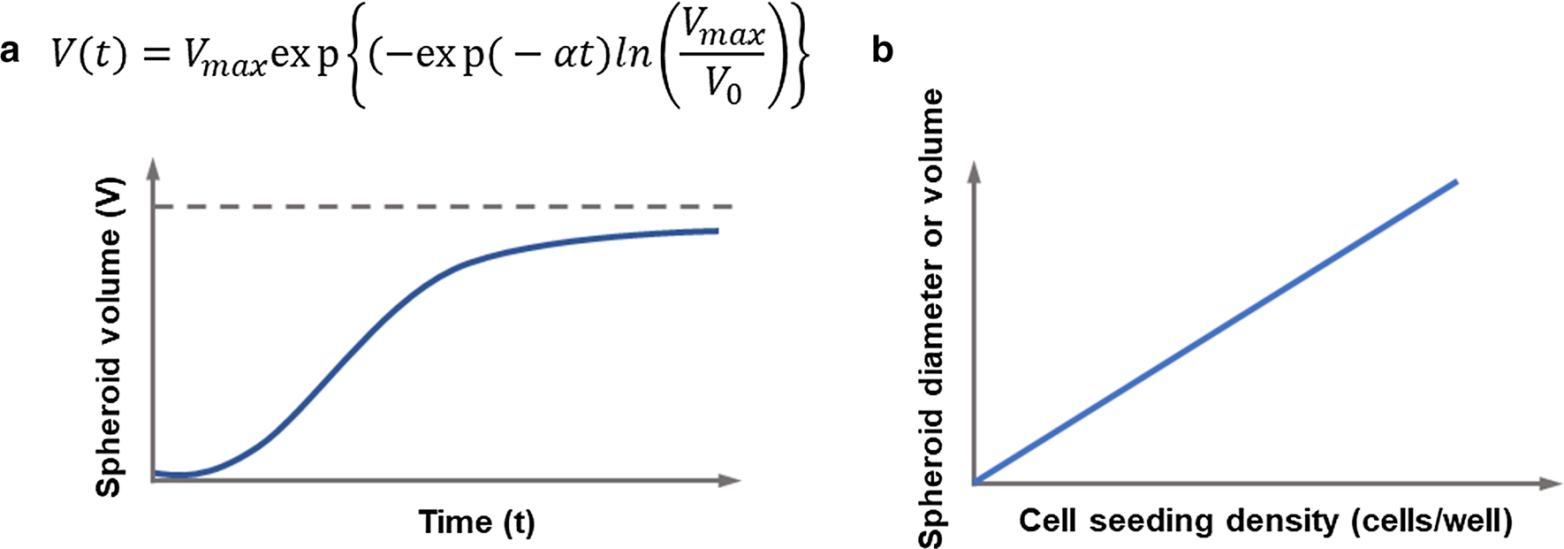
**a** Growth kinetics of MCTs as a function of time which follow the mathematical model suggested by Gompertz [64]. **b** Spheroid size as a function of the cell seeding density

The MCTs growth follows an S-shaped curve as a function of culture time with three distinct phases: an initial exponential phase, a linear phase, and a plateau (Fig. 3A). After the initial exponential phase, the spheroid grows rapidly for several days, and then the growth plateaus due to the increasing number of quiescent cells and the accumulation of necrotic cells inside [12, 56]. Several mathematical models have explained spheroid growth kinetics, such as exponential, logistic, and Gompertz models [65, 66]. Among them, the Gompertz model is frequently used to describe spheroid growth kinetics because it reportedly shows excellent agreement with experimental data for various tumor types [65–67]. The Gompertz model is given as follows [66]:

1 $$V\left(t\right)={V}_{max}\mathrm{exp}\left\{\left(-\mathrm{exp}(-\alpha t\right)ln\left(\frac{{V}_{max}}{{V}_{0}}\right)\right\}$$

in which *V*_0_ is the initial spheroid volume, *V*_*max*_ is the limiting volume, and *V*(*t*) is the volume at time *t*. *α* is the specific growth rate. This model predicts an approaching at the asymptotic volume of *V*_*max*_.

The MCTs size increases with increasing cell seeding density (Fig. 3b). A positive linear correlation between MCTs size and cell seeding density has been reported for MCTs of several cancer cell types, such as glioma cells (U251, U87) [30, 69], breast adenocarcinoma MCF-7 [30], and the mesothelioma cell line H2052 [70]. However, the increasing rates of MCTs size as a function of cell seeding density were all different. Other correlations between MCTs size and cell density have also been reported. The increasing rate of MCTs size is high at relatively small cell density but gets lower as the density increases, and then, it reaches a plateau [13, 71–74]. For example, the size of a breast cancer spheroid (T47D) was 200–300 µm at a cell density of 1 × 10^6^ cells/mL and increased to 250–300 µm with a seeding density of 3 × 10^6^ cells/mL [71]. For a seeding density of 3–10 × 10^6^ cells/mL, the mean diameter of the spheroid was almost the same, with a minor difference of 25 μm.

### How to form MCTs of uniform shape and size?

Although various techniques have been developed, it is still challenging to produce MCTs that are homogeneous in shape and size. There are several reasons why the generation of such MCTs is important for medical applications. First, it enables reproducible results in drug screening and achieving a meaningful level of tumor biology. Second, it provides a means to quantify treatment plans and estimate the impact of treatment uncertainty on the results. In most circumstances, compact spheroids are more resistant to the drug than aggregated cells, and smaller spheroids are more sensitive to both chemotherapy and radiotherapy [11, 75–80]. This is because the degree of drug penetration is poor where there are tight cell-to-cell adhesions, and the presence of hypoxic cells in larger MCTs may increase resistance to the therapy. And third, the mass production of homogenous MCTs enables high-throughput drug screening.

#### Form compact MCTs by adding additives

**Fig. 4:**
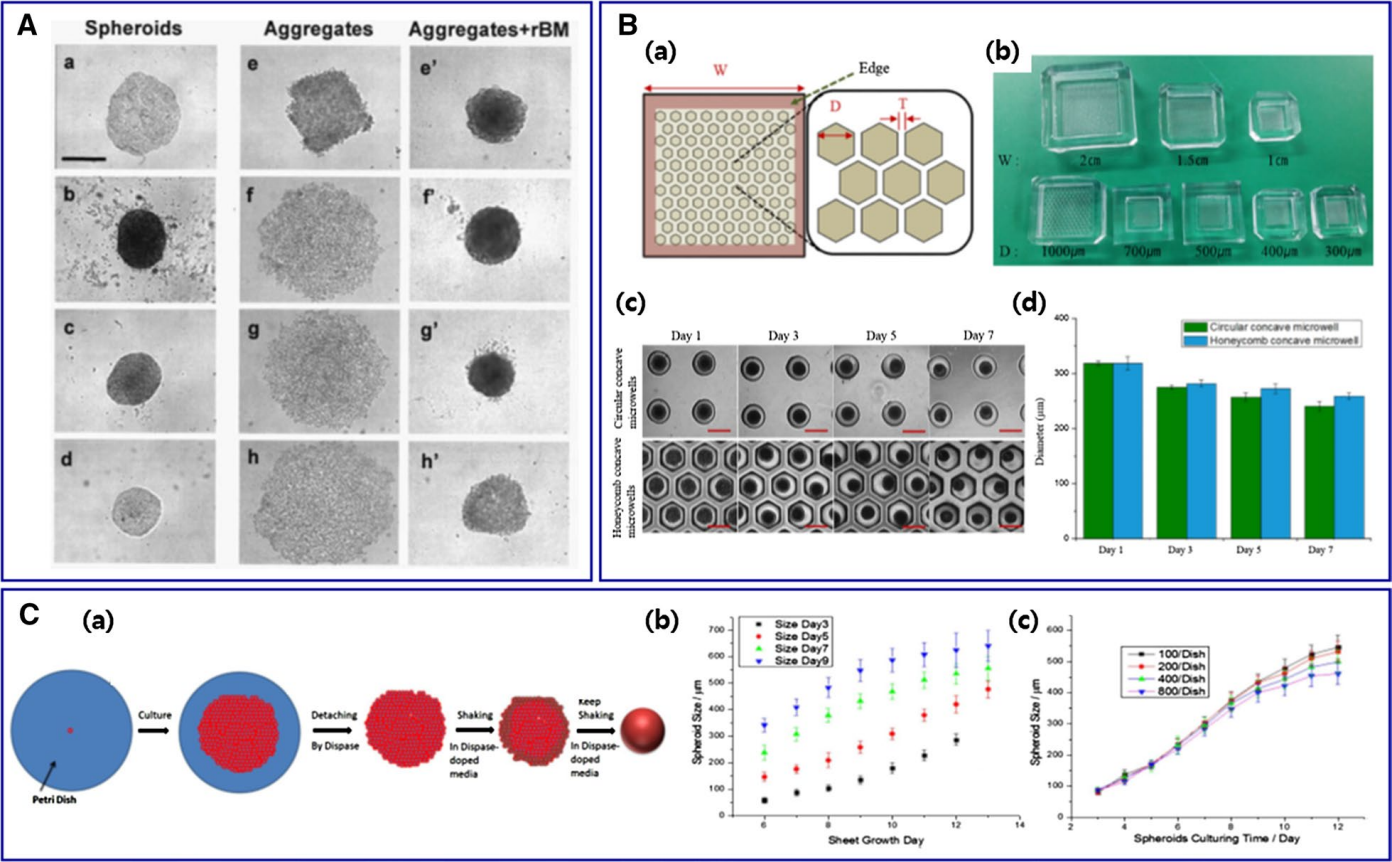
**A** Various morphologies of MCTs depending on cancer cell lines. Compact MCTs were generated with (a) MCF-7, (b) BT-474, (c) T-47D, and (d) MDA-MB-361. (e) MDA-MB-435S cells aggregated tightly but 3 cell lines of (f) MDA-MB-231, (g) MDA-MB-468, and (h) SK-BR-3 aggregated loosely. Adding 2.5% rBM yielded significant compaction (e'–h'). Bar: 500 μm. Reproduced with permission [22]. Copyright 2007, Demetrios Spandidos. **B** Honeycomb concave microwell. (a) Schematic diagram of a honeycomb concave microwell array (width [W], diameter [D], wall thickness [T]). (b) Various sizes of the honeycomb concave microwell chambers. (c) MCTs formation in the circular and honeycomb concave microwells. Bar: 500 μm. (d) The evaluation of hepatocyte spheroids in 2 different concave microwells [84]. Copyright 2016, Permits unrestricted use. **C** (a) Illustration of MCTs formation. (b) HCT-116 MCTs size as a function of sheet growth time. The sizes were recorded on different shaking days (days 3, 5, 7, and 9). (c) MCTs size as a function of culturing time with different initial cell seeding density [86]. Copyright 2018, Springer Nature

Several techniques have been introduced to generate compact MCTs with homogeneous sizes. As mentioned before, cell lines that express low intercellular junction proteins cannot form spheroids well. Adding of appropriate reconstituted basement membrane in the culture media can contribute to compact and circular spheroid morphology (Fig. 4A) [22, 59, 81–83]. Various additives, such as Matrigel, rBM, Geltrex®, and collagen, are suggested to support spheroid formation. In the presence of Matrigel, the breast cancer cell line (MDA-MB-231), which expresses low levels of E-cadherin, successfully generated well-defined 3D spheroids with uniform morphology, increased diameter, and good circularity [19]. The addition of 2.5% rBM encouraged cell-to-cell contact and resulted in the formation of compact spheroids with other breast cancer cell lines (MCF-7, BT-474, T-47D, and MDA-MB-361) [77]. The addition of Geltrex® under proper conditions also induced homogeneous and compact spheroids with SUM1315 and MDA-MB-231 [59].

#### Size control by microwell-based culture

Microfabrication of microwells has been widely employed to generate size-controlled spheroids. The microwells are conventionally fabricated using a micro-mold patterned by soft lithography and 3D printing technology (Fig. 4B) [19, 30, 36, 72, 80, 84–87]. This technique is simple, offers easy control over the well size, and is compatible with existing techniques. Various synthetic polymers are used in microwell fabrication, such as polymethylmethacrylate (PMMA), polystyrene (PS), polydimethylsiloxane (PDMS), and photopolymerized polyethylene glycol dimethacrylate (PEGDMA) [84]. The hydrogel formed by natural polymers, such as collagen, gelatin, alginate, and agarose, are also frequently adapted in microwells [72]. Since the cells are confined spatially and the microwells act as centers of cell attachment for self-aggregation, spheroids generated in microwells are more homogeneous in size compared to those generated using conventional techniques, such as liquid overlay and hanging drop techniques [85]. Besides, the size of spheroids can be easily controlled by varying microwell dimensions [80].

#### Other methods for uniform MCTs size

In addition, various methods have been suggested to get uniform-sized MCTs. The microprinting of MCTs using the aqueous two-phase system (ATPS) generates size-controlled HT-29 spheroids with an average diameter of 440 ± 24 µm [11]. Herein, ATPS comprises aqueous solutions of polyethylene glycol (PEG) and dextran (DEX). Cancer cells in the DEX phase nanodrop can form spheroids homogeneously because of the interfacial tension between PEG and DEX. Size-controlled MCTs can also be generated by shear flow under orbital shaking of media (Fig. 4C) [88]. Single cells seeded onto petri dishes can form cell sheets after a few days. The cell sheet is detached by dispase and then shaken in dispase-supplemented media. With the assistance of shear flow, the cell sheet yields spheroids. The spheroid size can be controlled by monitoring culturing and shaking times as spheroid size increases with increasing shaking and culturing times.

## Assessments

**Fig. 5:**
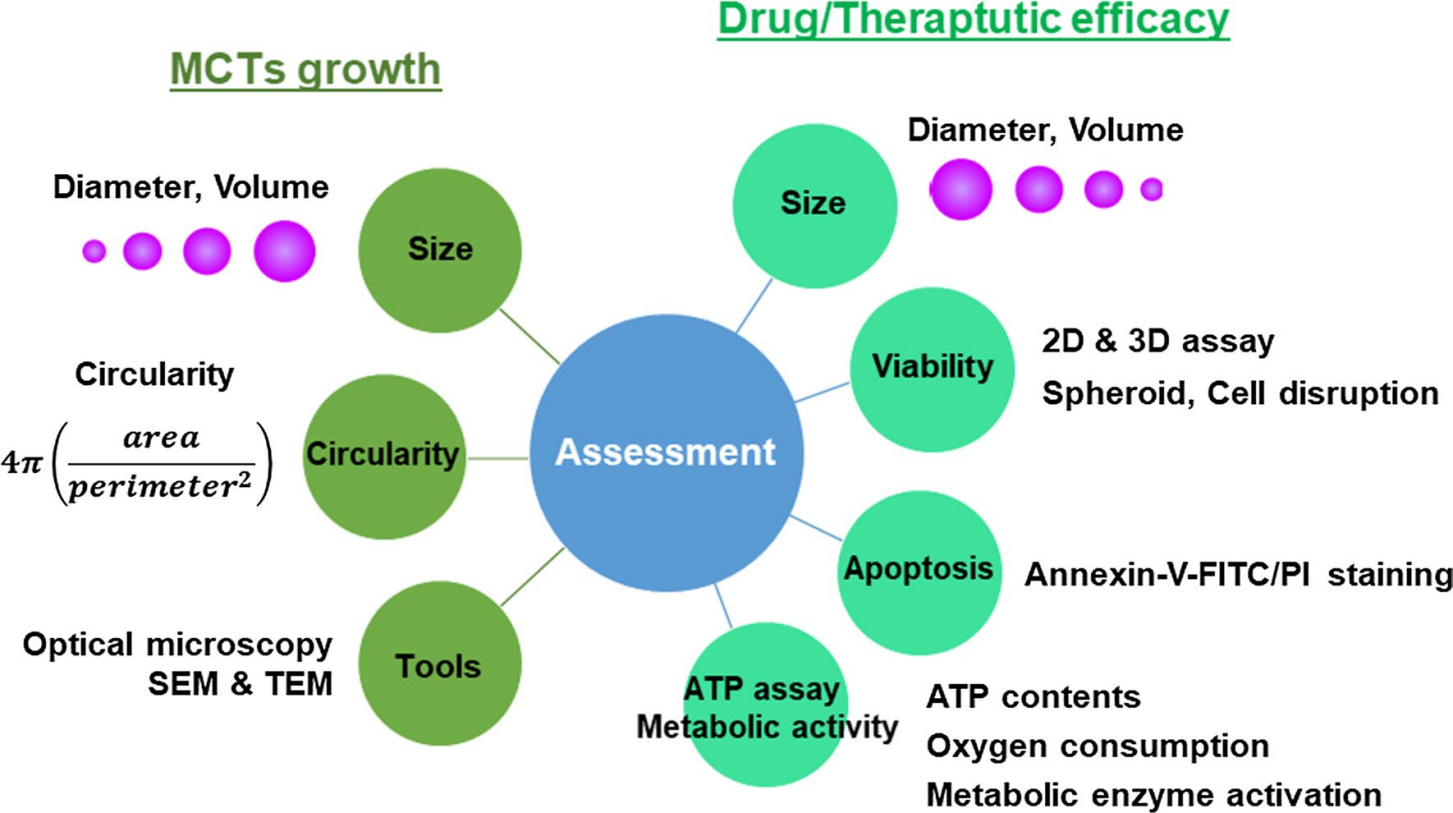
Two purpose of MCTs assessment and main criteria. MCTs' size and morphology and viability and apoptosis of cells in spheroids are measured to evaluate growth characteristics and drug efficacy of MCTs

The assessment of MCTs serves two purposes: (1) growth characteristics assessment and (2) drug and treatment efficacy assessment (Fig. 5). The application of MCTs requires detailed characterization of nature, growth kinetics, and response to chemo- and radiation therapy.

### Evaluation of growth characteristics

The two main features that characterize the growth of MCTs are shape and size. The shape of spheroids is determined by images taken by optical microscopy. Whether it is a perfect sphere or not is distinguished by optical microscopy [81]. The circularity of the spheroid is determined from the captured 2D optical microscopic image with a software program, such as Image J [56].

2 $$\mathrm{Circularity}=4\uppi \left(\frac{area}{{perimeter}^{2}}\right)$$

A circularity value ranges from 0.0 for the infinitely elongated polygon to 1.0 for a perfect circle. For example, the circularity of OVCAR8 spheroids ranged from 0.86 and 0.95 depending on the culture method [56]. In the case of a perfect sphere rather than an aggregation, the value of circularity increased over a few days (~ 5 days), indicating that the cells self-organized and formed a circular spheroid. Then, the circularity decreased for the remaining culture period due to cell proliferation at the spheroid periphery [30, 72, 81, 89].

The MCTs size is an important parameter to characterize the growth of MCTs. Conventionally, MCTs size is determined by measuring two orthogonal diameters from the optical microscopy image. Area and volume can be calculated using the diameter. It is necessary to accurately measure the spheroid size from microscopy images to produce reliable research results. Understanding the growth dynamics of spheroids depending on the diverse factors that contribute to MCTs growth, such as culture time, cell seeding density, and cell type plays a critical role in developing and improving treatment modalities.

### Evaluation of drug and therapeutic efficacy

Despite significant investments in cancer research and drug discovery, the complex nature and behavior of cancer cells make it extremely challenging to study them with a view to cure cancer. Recently, MCTs have been frequently considered to evaluate and predict tumor response to chemotherapy and radiotherapy because of their physiological similarities to in vivo solid tumors. Since MCTs respond differently to the therapy compared to 2D monolayer culture models, an appropriate evaluation method must be established using spheroids.

#### MCTs response to drugs and treatment

Evaluation of drug and therapeutic efficacy using MCTs has several characteristics. First, changes in the shape and volume of MCTs are the primary outcome showing therapeutic effects. As the treatment progresses, cell-to-cell and cell-to-matrix interactions are disrupted due to cytotoxicity, thus leading to disruption of cell aggregation [90]. As the cells at the edges fall apart, the shape of the sphere collapses. Consequently, the volume of spheroid decreases during the treatment in a dose-dependent manner [59, 91].

Second, the therapeutic effects depend on the compactness of MCTs. The cells in the aggregation are very sensitive to the treatment, whereas the cells in compact spheroids are not [56]. This can be correlated with the extracellular matrix content in MCTs. As mentioned before, compact spheroids possess a higher extracellular matrix content than aggregations, thus impeding drug delivery inside the spheroids. Therefore, we can get reliable results when spheroids of a uniform morphology are used to evaluate therapeutic effects.

Third, the therapeutic effects also depend on the MCTs size. Cells in small spheroids (< ~ 300 µm diameter) are sensitive to the treatment, whereas large spheroids (> ~ 500 µm diameter) show treatment resistance [75, 76, 78, 92]. This difference is related to the penetration depth of the drug or X-ray. As an example, doxorubicin (DOX), which is a commonly used chemotherapy drug, penetrates well into small MCF-7 spheroids but only to the outer few cell layers in larger spheroids at the same time [68]. Although the depth of penetration increases with time, the difference does not change. Since the therapeutic effect is very sensitively affected by the MCTs size, many studies have been focused on the formation of MCTs that are homogeneous in terms of size, as described in “Uniformity and reproducibility”, “Assessments”, “High-throughput platform”, “Conclusion and future prospects” sections.

#### High resistance to treatment

MCTs showed high resistance to most therapies, including chemo-, radio-, and phototherapy [72, 93–95]. Many studies have been reported for higher chemoresistance of cells in MCTs than in 2D monolayers. When the cells were treated by DOX for 3 days, the drug resistance of MCF-7 cells was 50 times higher in MCTs than in 2D culture: the IC_50_ value for spheroid culture was 23.2 μg/mL and that of 2D culture was 0.46 μg/ mL [72]. In the case of human lung carcinoma (A549), cells in MCTs exhibited about 6,600 times more resistance to vinblastine treatment than cells in monolayer [93]. The IC_50_ value of MCTs was 53 μmol/L and that of the monolayer was 0.008 μmol/L.

Under in vivo conditions, cancer cells in a solid tumor can acquire chemoresistance and radioresistance for several reasons: (1) Cancer cells can acquire the resistance through interaction with surrounding cells or with the ECM, such as collagen, laminin, and fibronectin [96]. Because stromal cells support the survival of cancer cells, the interaction between the cancer cells and the stromal cells increases treatment resistance [97]. (2) Densely packed cells interfere with the supply of oxygen into the tumors. This results in a gradient in oxygen concentration along with the tumors, and the presence of hypoxia inside the spheroids reportedly increases the chemoresistance of the cells [43]. (3) Nutrients like glucose and essential amino acids also have limited penetration toward the inside of tumors. The cells inside use glycolysis to survive, which results in increased production of CO_2_ and carbonic acid. The acidic microenvironment also causes inefficient drug delivery into the cancer cells [98].

The high resistance of MCTs to chemotherapy occurs similarly to in vivo solid tumors. (1) The penetration of the drug into the MCTs is limited by their diameter. The DOX penetrates well into small MCTs (2,000 MCF-7 cells per spheroid), but the penetration was restricted to the outer layer (~ 100 μm in depth) in large MCTs (8,000 MCF-7 cells per spheroid) [72]. Therefore, large MCTs show higher drug resistance than small MCTs. (2) Large MCTs of > 500 µm in diameter produce molecular gradients, such as nutrient, oxygen, pH, and metabolite, as mentioned before [11, 12]. The hypoxia condition in MCTs' inner zone causes high expression of P-glycoprotein and hypoxia-inducible factor 1 (HIF-1), which has been known to associate with drug resistance in various cancer cells [99]. (3) Drug resistance depends on the morphology MCTs. The drug can easily penetrate loosely aggregated spheroids, but it is challenging to penetrate compact spheroids, as mentioned before. Therefore, the resistance increases as the compactness of MCTs improved.

#### Effects of ECM on drug resistance

ECM is a highly complex fibrous construct composed of proteins (e.g., collagen, fibronectin, elastin) and polysaccharides (e.g., hyaluronan, glycosaminoglycan) [100]. The ECM serves as an essential supporter for tissues and regulates tissue development and homeostasis. ECM composition and mechanical properties significantly affect cellular functions such as cell growth, survival, migration, and differentiation [101].

The fibroblasts are a significant ECM source in both normal and malignant tissue; however, the ECM in tumorous tissue differs notably from that in normal tissues. Cancer cells produce substantial quantities of ECM during cancer progression, which has constituent ratios different from those from normal tissues. ECM proteins, such as laminin 5, hyaluronan, and TNC, and heat shock protein 47 are highly expressed in cancer cells [102, 103]. Increased collagen VI expressions accelerate cancer cell proliferation. Col5A2 and Col11A1 are highly expressed in invasive ductal carcinoma and trigger cancer cell proliferation. The expression of ECM genes and proteins also depends on the culture conditions [104]. The gene and protein expression profiles of cadherin and gap junction molecules were altered under 3D culture conditions. The mRNA and protein expression levels of N-cadherin were significantly higher than those of E-cadherin in early-stage HOSS1 MCTs formation; however, the opposite was observed in the late phase. E-cadherin protein expression was higher in MCTs than in 2D cultures due to the cell–cell contacts over the entire surface of the MCTs. Changes in the content, composition, and organization of the tumor ECM contribute to drug resistance. The increased expression of ECM proteins, such as collagen and fibronectin‐1, in MCTs contributes to establishing a chemoresistant environment for anticancer drugs, such as doxorubicin, gemcitabine, and docetaxel [104]. High ECM protein levels result in physical resistance to diffusional transport, and well-organized collagen fiber results in a stiff ECM, resulting in increased chemical protection [105].

### Issues of cell viability assay using MCTs

There are various assays to check the viability of cells, such as colorimetric, luminescence, and fluorescence assays. Nearly all of these assays have been optimized for use with 2D monolayer cell culture. Several 3D-specific assays have been developed and commercially available, such as Cultrex® 3D colorimetric and CellTiter-Glo luminescent assays [41, 106]. However, despite these specific assays, the analysis of cell viability of MCTs mainly relies on conventional 2D methods to date (Table 3).

**Table. 3.** Cell viability assays employed in the evaluation of drug efficacy using MCTs

Assay type	Culture model	Cancer cell	Drugs	Readout	References
CellEvent Caspase-3/7 Green	2D	Prostate cancer (VCaP, LNCaP)	MLN4924	Spheroid	[107]
LysoTracker Deep Red	2D	Prostate cancer (VCaP, LNCaP)	MLN4924	Spheroid	[109]
Annexin V-APC	2D	Breast cancer (MDA-MB-435S, MDAMB-468, MDA-MB-231, SK-BR-3)	–	Disassociation	[22]
MTT	2D	Breast cancer (MCF-7)	Tamoxifen	Disassociation	[97]
LDH	2D	Breast cancer (MCF-7)	Tamoxifen	Disassociation	[97]
AlamarBlue	2D	Lung cancer (H460, A549, H1650)	Cisplatin, Gemcitabine 5-fluorouracil, Camptothecin	Disassociation	[59]
	2D	Breast cancer (MCF-7)	Doxorubicin	Spheroid	[69]
Live/Dead	2D	Breast cancer (MCF-7)	Doxorubicin	Spheroid	[110]
	2D	Breast cancer (MCF-7)	–	Spheroid	[73]
Cultrex® 3D Colorimetric	3D	Lung cancer (A549)	4-HPR-HSA	Spheroid	[106]
CellTiter-Glo Luminescent	3D	Glioblastoma (U-87 MG, KNS42) Oral squamous (LICR-LON-HN4) Breast carcinoma (MDA-MB-231)	HSP90 chaperone inhibitor PI3 kinase/mTOR inhibitor PLCg inhibitor	Spheroid	[41]

In viability assays, the fluorescence signal can be readout from disaggregated cells or whole spheroids. Disruption of MCTs and analysis of cell lysates or suspensions is the more commonly used approach. Like the 2D culture model, the signal from disrupted cells readout by microplate readers. In this case, traditional plate reader reading may miss multiple aspects of spheroids, including morphological information. Therefore, analysis methods that involve imaging of MCTs without disruption are preferred. However, effective analysis of MCTs using conventional imaging systems is also challenging due to time-consuming image acquisition and complex image processing, among other concerns [106].

Since cells in MCTs respond to drugs differently from cells in a 2D monolayer, protocol optimization for viability and apoptosis assays using MCTs might be required. First, incubation time with assay reagents should be determined depending on the MCTs size. To ensure sufficient penetration of assay reagents into spheroids, the reagent absorption should be optimized before getting experimental results [41]. Second, the assay should be optimized depending on the compactness of MCTs. The differences in compactness of MCTs would lead to different penetration of assay reagents. Therefore, optimum assay conditions should be confirmed with MCTs of different cell types and generation methods. In general, more massive and more compact MCTs require more prolonged incubation with assay reagents. Finally, for quantitative analysis, the fluorescence signal from MCTs should be interpreted considering their size and structural characteristics. If a strong fluorescence signal from large MCTs is accepted without accounting for its size, it could lead to inaccurate conclusions.

### Apoptosis and ATP assay

The apoptosis of cells in MCTs can be analyzed using flow cytometric detection by annexin V/PI staining, which is the method of confirmation used in 2D monolayer cells [72, 107, 108]. Before staining, the MCTs are disaggregated into a single-cell suspension using enzymatic dissociation. Complete dissociation of the cells without affecting their viability is critical for the accurate detection of apoptosis in MCTs. Cellular viability in MCTs can also be assessed by measuring the intracellular ATP content. The heterogeneous physical characteristics of MCTs, such as size, composition, and penetration depth, pose challenges in performing ATP assays; however, a suitable method for MCTs has been developed that optimizes the detergent composition and lysis conditions [93, 111, 112]. ATP is conventionally detected using bioluminescence, which offers robust, sensitive, and scalable high-throughput screening. The metabolic activity, such as oxygen consumption and metabolic enzyme activation, is also employed to assess MCTs viability [93, 113, 114].

### Biophysical property of MCTs

In vivo, solid tumors are complex tissues containing cancer and stromal cells, ECM, blood vessels, and lymphatic vessels. Their physical properties are highly dynamic and evolve during tumor growth and progression. The tumor cells experience constant physical stimuli that affect tumor biology, including hydrostatic pressure, shear stress, compression, and tension [115]. Compressive stress reduces the cancer cell proliferation rate, induces apoptosis, and alters the expression of specific genes related to the invasive and metastatic potential of cancer cells [116–120]. Compression of fibroblasts in a tumor accelerates the production of ECM components, increasing tumor stiffness [115, 121].

Several factors cause stress, including both internal and external. The localized proliferating cells on the outer layer and necrotic cells in the core generate a cellular flow from the spheroid rim toward its core. This flow creates shear stress within the tumor [122, 123]. The stiff ECM applies compressive stress on the cells [124, 125]. Plasma leakage from blood vessels into the tumor interstitial space can increase the hydrostatic pressure inside the tumor [115].

Various techniques have been attempted to assess the physical properties and the stress that the tumor experiences or generates. Tumorous tissue exhibits significantly different elasticity than normal tissue. The elastic modulus of a human brain tumor is about 35 kPa, whereas that of normal brain tissue is 2.0–6.0 kPa [126]. Breast cancer tissue also shows a higher elastic modulus (10.0–42.0 kPa) than normal breast tissue (approximately 3.25 kPa) [127]. The elastic modulus of T24 (epithelial bladder cancer cells) MCTs was determined basis diameter variations using atomic force microscopy (AFM; 113, 226, 235, 250 μm); no significant differences in elasticity were observed [128]. In a study, the mechanical stress in CT26 (colorectal cancer cells) MCTs was measured using a pressure sensor made of polyacrylamide microbeads; stress increased toward the MCTs core and was unevenly distributed [129]. The contractile forces exerted by MCTs can be determined by tracking the deformation of the collagen matrix using bright field time-lapse microscopy [130]. However, owing to the limitations of contractile force measurement techniques, computer simulations were used to explain the physical forces that cause matrix deformation. Assuming a negative hydrostatic pressure, the simulation predicts that the MCTs' core causes the collagen matrix's most severe deformation. The extent of deformation decreases toward the outside of the MCTs.

## High-throughput platform

Despite several advantages of MCTs, its extensive use for drug screening is still limited because the traditional MCTs forming system takes a long time to culture and produces MCTs of various sizes. The application of MCTs in high-throughput drug screening requires establishing a rapid generation of homogeneous MCTs and a well-established screening procedure. Recent advances in microfluidic technology have contributed significantly to the development of high-throughput screening systems using MCTs.

### MCTs generation in microfluidic device

Microfluidic technology refers to the manufacture of miniaturized devices that include chambers and channels where fluid flow is geometrically limited [131]. Microfluidic technology has been considered a powerful tool for various biological research fields, such as tissue engineering and drug screening. The microfluidic device offers precise manipulation of cells at the micro or nanometer scale as well as precise handling of microenvironments in terms of pressure and shear stress on the cells [132]. The device can also provide gradients of chemical concentration and continuous perfusion with minute liquid volumes. The use of microfluidics in MCTs culture has been suggested in various versions.

#### Microwell-based microfluidics

**Fig. 6:**
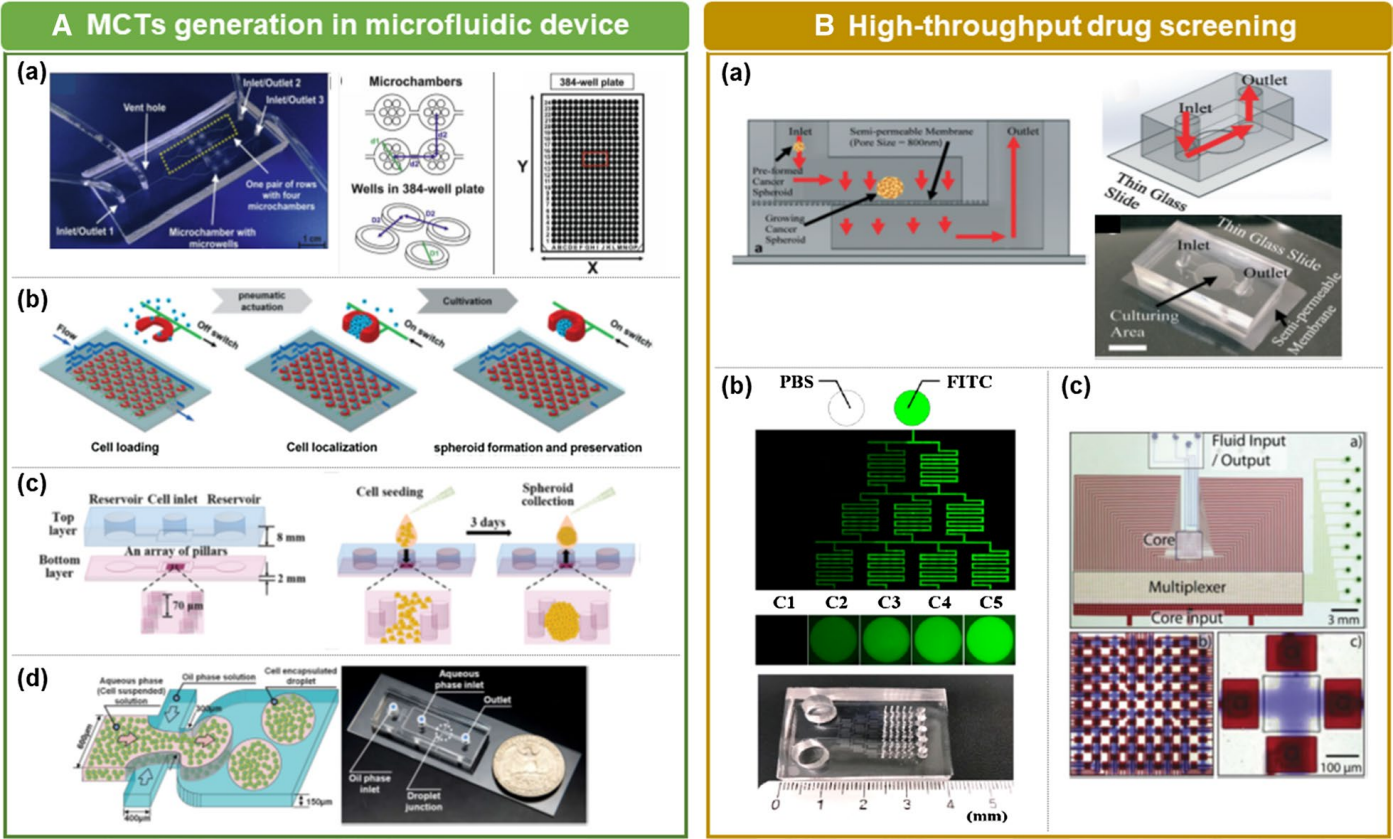
**A** MCTs generation in a microfluidic device. (a) Schematics of a microchip containing of 4 rows of microchambers that contain 7 microwells [130]. Copyright 2017, Elsevier. (b) A schematic diagram of the pneumatic microstructure array and its operating principle [141]. Copyright 2015, The Royal Society of Chemistry. (c) A schematic diagram of the microfluidic pillar array with cell seeding and collection processes [143]. Copyright 2018, The Royal Society of Chemistry. (d) Schematic and optical images of droplet-based microfluidic systems for MCT fabrication [53]. Copyright 2018, Elsevier. **B** High-throughput drug screening. (a) Microfluidic device for rapid tumor spheroid growth consisting of a semi-permeable polycarbonate membrane [52]. Copyright 2019, The Royal Society of Chemistry. (b) The microfluidic device generates a concentration gradient of fluorescein isothiocyanate (FITC). The fluorescent images of channels and concave microwells (C1–C5) were taken 16 h after an injection of phosphate-buffered saline (PBS) and FITC [133]. Copyright 2018, Permits unrestricted use. (c). The architecture and operation of the software-programmable microfluidic platform [154]. Copyright 2018, The Royal Society of Chemistry

Microwell-based microfluidic devices are considered the most suitable candidate for studying drug efficacy in high-throughput screening techniques (Fig. 6A (a)) [133]. The device is specified with a number of microwells connected to a loading chamber through a microchannel [134–136]. The cells are delivered from the loading chamber to the microwell and then self-aggregate to form MCTs over time. Each microwell is evenly filled with a cell suspension to obtain a MCTs of uniform size. Therefore, mass production of size-controlled MCTs can be achieved using the microwell arrays.

One of the advantages of microwell-based devices is compatibility with existing laboratory technology and instrumentation [137]. With accumulated know-how for a long time in this regard, microwell plates have become a standard tool for various applications of the 2D monolayer culture model, including cell culture, sample storage, sample filtration, assay, and drug screening. Microwell plates are typically made of plastic or glass and are available in multiple formats, including 24-, 48-, 96-, 384-, 864-, and 1,536-well plates. A microplate reader is used to detect biological or chemical signals from the microwell plate. Thus far, various versions of microplate readers have been developed and customized. If the size and the arrangement of the microwell in the microfluidic device is matched with the conventional microwell plates, it can easily ensure compatibility with all established technology and instrumentation [133, 138]. This compatibility is critical for the commercialization and automation of the microwell-based microfluidic device.

Meanwhile, the fabrication process of microwell-based microfluidic devices is relatively complicated, labor-intensive, and time-consuming. Typically, microfluidic devices are fabricated by soft lithography and etching in two steps of master fabrication and PDMS replica molding [139]. To overcome this disadvantage, simple fabrication methods using 3D printer have been suggested as 3D printing does not require special instruments and can fabricate the mold in a single step [28].

#### Traps in U-shaped microstructures

Trapping cells in microstructures also provides a massive and high-throughput platform. Cells can be trapped by active and passive methods. Active traps use external power such as electrical or optical sources to capture the cells, whereas passive traps do not require any external source [140–142].

The use of U-shaped microstructures integrated into the microfluidic device is a passive approach using hydrodynamic traps. Typically, the culture chamber of the MCTs is formed by bonding a PDMS device to a glass substrate, wherein a number of U-shaped traps are arranged [7, 143]. When suspended cells are loaded into the chamber, the cells are hydrodynamically captured by the U-shaped trap. Excess cells are expelled with the fluid after loading the cells. This device can simultaneously generate a large number of spheroids with a narrow size distribution. The spheroid size and shape are influenced by the flow rate of the fluid. Higher flow rates are better for confining the cells, thus leading to a more uniform and firmer spheroid growth [7]. Furthermore, the MCTs growth rate is faster under higher flow rates. If the U-shaped traps are structurally deformed by gas pressure, a reversible operating platform can be achieved in terms of the spheroid being positioned and released from the device. When gas pressure is applied to the U-shaped trap, it transforms into a structure that can capture cells well, and when the air pressure is blocked, it returns to its original form and releases the spheroids (Fig. 6A (b)) [144]**.**

#### Confining with micro pillar array

The use of a micropatterned surface and low-adhesion plates in microfluidic channels enables the formation of size-controllable MCTs. The bottom layer of the microchannel contains a pillar array with a specific distance and a rectangular wall surrounding the pillars in which cells gather (Fig. 6A (c)) [145, 146]. Since micropatterned pillar arrays laterally confine the cells, they may aggregate and form a spheroid uniformly. The pillar arrays provide mechanical support and confined space by which the spheroid size is controlled [136]. The distance between pillars positively affected the MCTs diameter. A microfluidic device with pillar array can mass-generate MCTs with a relatively uniform size compared with conventional spheroid culture technology. However, there are limitations in expanding the MCTs volume.

#### Droplet-based microfluidics

Micron-size droplets formed by interfacial tension differences between two different solutions are used for mass production of MCTs (Fig. 6A(d)) [55, 147]. Cells are encapsulated in the droplet and then aggregate because they cannot settle down on the surface and form a single spheroid structure. The droplet diameter and generation yield are controlled by fluid flow velocity [145, 148]. As the flow velocity increases, the diameter decreases, and the generation yield increases. Although the spheroid size can be controlled by the number of encapsulated cells in a single droplet, forming a large-sized spheroid remains challenging [149].

### High-throughput drug screening

Drug development is a long, complex, and expensive process that involves significant basic research and preclinical evaluation. Microfluidic device combined with MCTs has several advantages in drug screening, such as in vivo-like environment, high analytical throughput, enhanced sensitivity, and facile parallelization through multiplexing.

#### MCTs culture for a long time

In a drug screening employing MCTs, it is essential to stably supply oxygen and nutrients for a considerable time and remove cellular waste products to maintain a similar environment as in vivo conditions. The perfused system of the microfluidic device is highly beneficial in preserving steady-state environments. For continuous nutrition supply, the microfluidic device was fabricated with upper and lower channels separated with a semipermeable membrane (Fig. 6B(a)) [54]. The MCTs can easily settle down on the semipermeable membrane and the uniformly distributed nano-sized pores in the membrane, which allows the transport of media and waste products. The MCTs individually entrapped in the microfluidic channels can be shielded from the shear stress caused by fluid flow [70, 150]. Therefore, the MCTs can grow stably, and the drug effects can be monitored for more than a week. Fluid perfusion in the device is generally performed using tubes and external pumps. The perfusion can also be achieved by gravity-driven flow without external equipment [151]. The gravity-driven flow generated by automated device tilting and the perfusion system offers simple medium exchange and liquid sampling.

#### Drug combination screening

Combination chemotherapy, which refers to the use of more than one anticancer drug at a time to treat cancer, has been widely applied for many types of cancers. Using a combination of drugs increases therapeutic efficacy because the different drugs affect cancer cells at different points in the cell cycle [152–154]. In general, the process of finding an effective drug combination is a time-consuming task that requires many replicates to screen for different concentrations and combinations of drugs. Microfluidic devices are beneficial for the screening of drug combinations, and various designs have been proposed (Fig. 6B(b)) [136, 142, 155, 156].

For example, a microfluidic channel with an 8 × 8 chamber array and two concentration gradient generators with two micropumps can produce 64 different combinations at once [155]. The device has two sets of reservoirs, and each reservoir can load an anticancer drug and a sensitizer separately. As the anticancer drug and sensitizer pass through the micropump, they are mixed in eight different concentrations by gradient mixer. Consequently, 64 different combinations are generated in 64 chambers from two sets of reservoirs. The microfluidic device, by varying channel size, enables a logarithmic mixing ratio gradient between two drugs [142]. In a device comprising five stages of microfluidic channels, the number of channels in each stage increased from 3 to 7, and the channel size was changed. The different channel size causes a different splitting ratio of the flow at each stage due to the different flow resistance. Thus, the increased channel size from one side to the other creates a nonlinear concentration gradient in the flow. This method allows 1,032 drug efficacy screening experiments to be performed with a single screening chip for eight drug combinations.

#### Scaling and automation for high-throughput

The conventional drug screening is performed manually and requires a skilled operator, making it expensive and not suitable for high-throughput screening. A successful drug efficacy screening and/or validation system requires the system to be robust, reliable, and compatible with automated high-throughput screening platforms. The microfluidic-based drug screening system enables the automation of many operations, such as sorting, positioning, monitoring, and drug delivery. The software-programmable microfluidic device enables automated process in various steps, including microfluidic display, fluid metering, and active mixing of compounds (Fig. 6B (c)) [157]. In addition, automated analysis software allows MCTs in microfluidic channels to be monitored in a high-throughput manner to determine their spherical shape and size [158].

## Conclusion and future prospects

For decades, numerous 3D models have been suggested in cancer research, which is mainly based on the MCTs model, organotypic slices of cancer tissue, and multilayered cell cultures [159–161]. Continuous progress in MCTs research has improved the diversity, fidelity, and capacity of MCTs culture models, and the MCTs culture system can now be commercially developed. The microwell-based culture system provides an easy way to generate a large number of MCTs, and the optimized culture medium increases the success rate of MCTs formation. Since 2000 the reports on MCTs research have increased dramatically. In particular, various methods and conditions have been proposed to use MCTs for drug screening. Unlike 2D monolayer models, MCTs exhibit considerable drug resistance due to their structural characteristics being similar to in vivo solid tumors, and therefore, the MCTs can be considered a more suitable drug screening model.

However, for MCTs to be the preferred culture model for drug screening during preclinical stages, the formation of uniformly sized spheroids and reproducibility must be ensured. Uniformity and reproducibility are considered the essential factors of MCTs generation because high-throughput drug screening platforms cannot be established with heterogeneous MCTs. Conventionally, the uniformity and reproducibility of MCTs evaluate based on their size, including diameter and volume. According to this criterion, various methods and techniques developed so far have achieved considerable success. However, since the MCTs size can distort the evaluation results when the MCTs density is different, it seems necessary to develop a new physical quantity, such as mass, that is more robust and easier to evaluate.

Patient-derived MCTs or organoids may offer robust preclinical drug-screening platforms to identify effective cancer therapy for individual patients. The patient-derived MCTs or organoids can provide valuable information about individual tumors because they structurally and functionally recapitulate the original tumor characteristics [162, 163]. They retained their original tumor characteristics such as glucose consumption, lactic acid production, HIF1a levels, and oxidative stress and did not show significant changes in gene expression profiles [164]. Immunohistochemical staining of breast cancer MCTs derived from surgical samples of human breast cancer tissue reveals a heterogeneous mixture of cellular components within spheroids, including epithelial markers (PanCK), fibroblast markers (Vimentin), and breast cancer-specific markers (ER, PR, Her2, Mammaglobin, GATA) [165]. Several patient-derived MCTs or organoids have been established, including prostate cancer, colorectal cancer, lung cancer, and pancreatic cancer [166–168]. However, their clinical application is hampered by technical difficulties. The primary culture of cancer cells can be challenging due to low cancer cell viability and potential contamination by host cells [169]. And supplements such as growth factors, minerals, vitamins, and hormones are required to produce patient-derived MCTs or organoids [170].

For several decades, 2D monolayer cultures have been the primary cancer research and drug screening model because they are easy, low-cost, and highly reproducible. However, since they cannot reproduce the real complexity and 3D structure found in the in vivo solid tumor, a 3D culture model will replace them in the near future. When the current issues concerning MCTs are solved and further improved, including vascularization and immune system components, it will be possible to promote the establishment of a platform applicable to anticancer drug search and screening by extracting valid biological information from 3D models.

## Data Availability

All data needed to evaluate the conclusions in the paper are present in the paper and/or the Supplementary Materials. Additional data related to this paper may be requested from the authors.
